# Electroconvulsive therapy modulates loudness dependence of auditory evoked potentials: a pilot MEG study

**DOI:** 10.3389/fpsyt.2024.1434434

**Published:** 2024-08-12

**Authors:** Michael Dib, Jeffrey David Lewine, Christopher C. Abbott, Zhi-De Deng

**Affiliations:** ^1^ Fischell Department of Bioengineering, University of Maryland, College Park, MD, United States; ^2^ The Mind Research Network, University of New Mexico, Albuquerque, NM, United States; ^3^ Department of Psychiatry and Behavioral Sciences, University of New Mexico School of Medicine, Albuquerque, NM, United States; ^4^ Computational Neurostimulation Research Program, Noninvasive Neuromodulation Unit, Experimental Therapeutics and Pathophysiology Branch, National Institute of Mental Health Intramural Research Program, National Institutes of Health, Bethesda, MD, United States

**Keywords:** electroconvulsive therapy, loudness dependence of auditory evoked potentials, major depressive disorder, magnetoencephalography, serotonin

## Abstract

**Introduction:**

Electroconvulsive therapy (ECT) remains a critical intervention for treatment-resistant depression (MDD), yet its neurobiological underpinnings are not fully understood. This pilot study aims to investigate changes in loudness dependence of auditory evoked potentials (LDAEP), a proposed biomarker of serotonergic activity, in patients undergoing ECT.

**Methods:**

High-resolution magnetoencephalography (MEG) was utilized to measure LDAEP in nine depressed patients receiving right unilateral ECT. We hypothesized that ECT would reduce the LDAEP slope, reflecting enhanced serotonergic neurotransmission. Depression severity and cognitive performance were assessed using the 24-item Hamilton Depression Rating Scale (HDRS_24_) and the Repeatable Battery for the Assessment of Neuropsychological Status (RBANS), respectively.

**Results:**

Contrary to our hypothesis, findings indicated a significant increase in LDAEP post-ECT (*t*_8_ = 3.17, *p* = .013). The increase in LDAEP was not associated with changes in depression severity or cognitive performance.

**Discussion:**

The observed increase in LDAEP suggests a more complex interaction between ECT and neurobiological systems, rather than a direct reflection of serotonergic neurotransmission. Potential mechanisms for this increase include ECT’s impact on serotonergic, dopaminergic, glutamatergic, and GABAergic receptor activity, neuroplasticity involving brain-derived neurotrophic factor (BDNF), and inflammatory modulators such as TNF-α. Our results highlight the multifaceted effects of ECT on brain function, necessitating further research to elucidate these interactions.

## 1 Introduction

Major depressive disorder (MDD) remains a pervasive global health challenge, affecting millions worldwide and ranking among the leading causes of disability. MDD leads to substantial healthcare costs and contributes heavily to the overall disease burden (1). Despite the widespread use of antidepressant medications, many patients do not achieve sustained relief. As an alternative, neuromodulation therapies such as electroconvulsive therapy (ECT) play a vital role. ECT is a well-established intervention that demonstrates exceptional efficacy in multiple psychiatric disorders, including MDD. It involves the administration of electrical currents, either unilateral or bilateral electrode placements on the patient’s head, to induce a brief, controlled seizure. Ultimately, this process is thought to elicit reorganization of key cortical networks involved with mood and cognition. However, ECT can cause significant adverse effects, such as memory loss and confusion, rendering ECT to be reserved for severely treatment resistant patients (2, 3). Identifying the specific neurophysiological changes induced by ECT could lead to the development of safer and more effective treatments. While the optimal stimulation methods and parameters are still being investigated, ECT remains essential for managing treatment-resistant depression.

More than eight decades have passed since its introduction as a clinical intervention, yet the precise neurobiological mechanisms underpinning ECT’s therapeutic effect remain elusive. Current research suggests that ECT’s benefits are likely achieved through multiple mechanisms. These include, but are not limited to, changes in neurotransmitter transmission, enhancement of neurotrophic and neuroplastic activities, modulation of cortical networks, reduction of neuroinflammation, and regulation of the endocrine system (3–10). Given the historical precedence of the monoaminergic theory of depression, a plethora of studies in both animals and humans have sought out to determine whether ECT’s efficacy is related to changes in serotonergic activity (11, 12). While the evidence remains inconclusive, ECT appears to have some notable effect on serotonergic neurotransmission (3). A significant challenge in this area is that peripheral biomarker measurements do not reliably reflect neurotransmitter levels in the brain. Advanced neuroimaging techniques can be employed to gather insight into the effect of treatments such as ECT on neurotransmitter activity.

Loudness dependence of auditory evoked potentials (LDAEP) is a method used to measure the response of cortical potentials to variations in the intensity (i.e., loudness) of auditory stimuli. LDAEP is typically assessed using electroencephalography (EEG). The relationship between stimuli loudness and evoked potential amplitudes in the primary auditory cortex has been suggested as an indicator of serotonergic neurotransmission (13). Serotonin is thought to play a role in auditory processing, as layer IV of the primary auditory cortex is densely innervated with serotonergic fibers originating from the raphe nucleus (14, 15). Initial studies in animal models reported that higher serotonin activity was correlated with less dependence on stimulus intensity (i.e., similar amplitudes in cortical evoked potentials regardless of loudness). Conversely, lower serotonin activity was correlated with loudness-dependent changes in evoked potential amplitudes (13, 16). Furthermore, LDAEP has been proposed to be a protective mechanism in auditory processing, which helps prevent overstimulation and excitotoxicity (17). Within the primary auditory cortex, the neurobiological mechanisms of a reduced LDAEP being associated with high serotonin activity is proposed to rely on serotonergic modulation of cortical excitability. This modulation occurs indirectly via GABA-ergic interneurons, which express excitatory 5-HT2A receptors, and directly via pyramidal cells, which express both excitatory 5-HT2A and inhibitory 5-HT1A receptors (13, 16, 18, 19).

An abundance of literature now exists exploring the relationship between the LDAEP and other neurotransmitter systems and biomarkers (20, 21). While evidence for the LDAEP’s relationship with serotonin is controversial, it is clear serotonergic activity serves a critical role in the functioning of the primary auditory cortex (20, 22–26). Numerous studies provide robust support for the influence of serotonergic activity on neuronal functioning across auditory processing pathways (27–30). Notably, a recent positron emission tomography (PET) study on the molecular mechanisms underlying the LDAEP reported that this biomarker is strongly and positively correlated with 5-HT1A binding in the temporal cortex, specifically in the location of the primary auditory cortex (15).

Additionally, studies have demonstrated that serotonin plays a crucial role at the intersection of psychiatric disorders and auditory conditions, including tinnitus and hearing loss (27, 28). In particular, MDD has been reported to be associated with impaired auditory processing. Studies indicate that deviations in serotonergic activity are evident in the auditory cortices of individuals with depression compared to controls (25, 31–34). For example, increased 5-HT1A binding and decreased 5-HT2A binding specifically within the primary auditory cortex in depressed patients has been reported (25). Moreover, treatments for depression, including ECT, have been shown to have a significant effect on auditory processing, demonstrated via increased activity, excitability, and intrinsic connectivity within the auditory cortices (31, 32, 35–37). Additionally, ECT has shown a pronounced impact on auditory evoked potentials, further underscoring the complex interplay between serotonergic modulation and auditory functions in psychiatric contexts (38, 39).

Given the efficacy of serotonergic agents such as selective serotonin reuptake inhibitors, and more recently psychedelics like psilocybin, in the treatment of depression, it is likely regulation of this monoamine system serves a pivotal role in ECT’s efficacy (40). Studies show mixed results regarding ECT’s impact on serotonergic receptors. For instance, some reports indicate that electroconvulsive stimuli result in decreased binding and activity of both 5-HT1A and 5-HT2A receptors (12, 41–43). However, other studies reveal no change in 5-HT1A activity, an increase in 5-HT2 activity, and enhanced serotonin transporter (SERT) receptor levels following ECT (44–47). Despite these discrepancies, there is broad consensus that ECT has a robust impact on serotonergic receptors in the treatment of multiple psychiatric disorders (8, 12, 48, 49).

Conventional LDAEP studies typically employ EEG. A 1982 study by Hari et al. compared simultaneous magnetic (AEF) and electrical (AEP) responses to auditory tones, demonstrating that AEF measured by MEG are highly comparable to AEP measured by EEG for short interstimulus intervals (*<* 4s) (50). MEG can offer better spatial resolution than EEG, particularly for superficial cortical sources (50). This comparison provides a foundational basis for the application of MEG in auditory evoked potential studies. Furthermore, subsequent studies have confirmed the reliability and validity of using MEG to measure LDAEP (51).^1^

This study utilizes high-resolution magnetoencephalography (MEG) to measure cortical activity and determine the LDAEP in individuals before and after ECT. The primary aim is to explore the changes in the central serotonergic neurotransmission attributable to ECT, by analyzing variations in LDAEP. We hypothesize that ECT will decrease LDAEP, indicative of enhanced serotonergic neurotransmission. This approach not only promises to deepen our understanding of the neurochemical environment in patients undergoing ECT but also sheds light on the neurobiological mechanisms that underpin ECT’s effectiveness.

## 2 Methods

Study participants and ECT treatment Ethical approval was obtained from the Human Research Protections Office at the University of New Mexico (UNM) before study initiation. The research was conducted in full compliance with the ethical standards outlined in the Declaration of Helsinki. Patients were recruited from the UNM Mental Health Center’s inpatient and outpatient services. All patients either had the decisional capacity to consent or, where necessary, provided assent with a surrogate decision-maker giving formal consent. All patients completed a full course of electroconvulsive therapy (ECT) using the ultra-brief pulse width, right unilateral electrode placement as previously described (52). During the initial session, the seizure threshold was determined using a dose titration method, which then guided the dosage for subsequent treatments. Specifically, the stimulus dosage was set at six times the threshold. Treatments were administered thrice weekly, and continued until an adequate clinical response was achieved or a decision was made to cease treatment due to non-response.

### 2.1 MRI

All MRI scans were conducted using the 3-Tesla Siemens Trio scanner at the Mind Research Network (MRN). High-resolution T1-weighted structural images were acquired using a 5-echo MPRAGE sequence with the following parameters: echo times (TE) of 1.64, 3.5, 5.36, 7.22, and 9.08ms; repetition time (TR) of 2.53s; inversion time (TI) of 1.2s; a flip angle of 7°; a single excitation; a slice thickness of 1mm; a field of view of 256mm; and a resolution of 256×256. Structural MRI preprocessing and the delineation of structural images were conducted using FreeSurfer 4.5.0 software (https://surfer.nmr.mgh.harvard.edu) (53).

### 2.2 MEG acquisition and data processing

**Figure 1:**
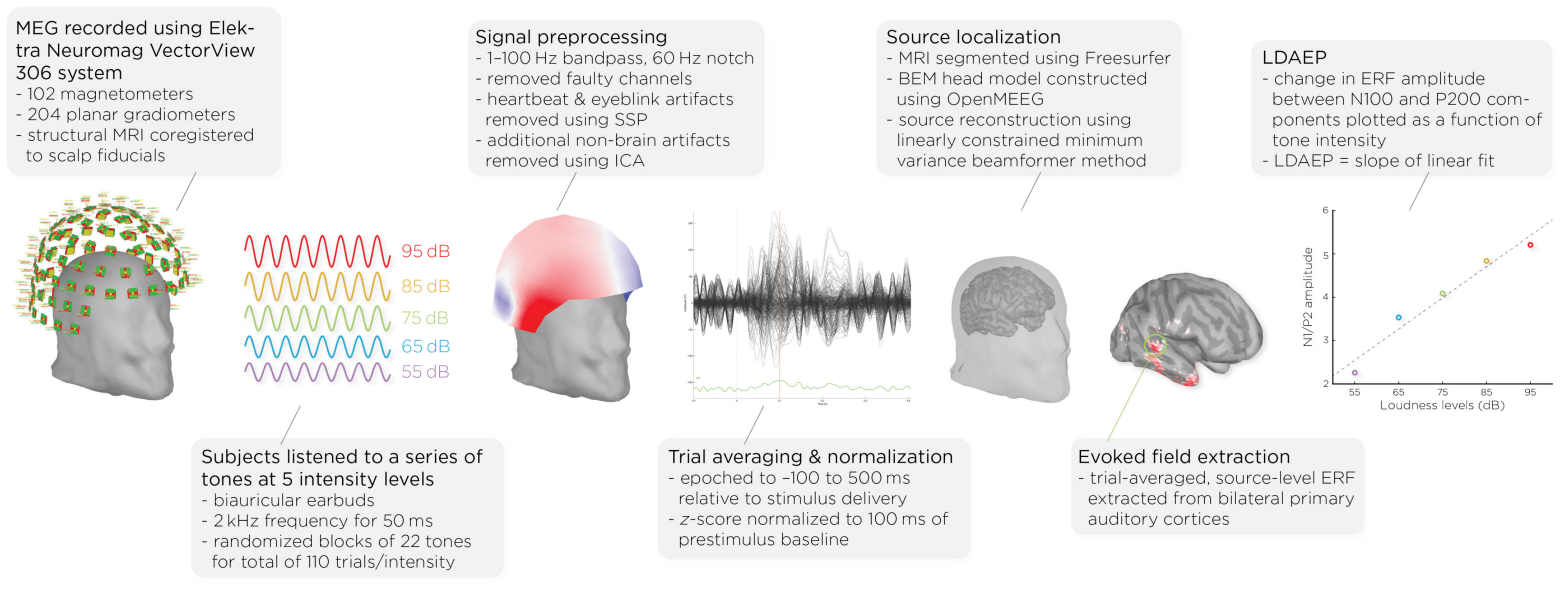
Workflow of MEG acquisition and data processing, detailing the steps from signal acquisition to data analysis. It shows the MEG setup, signal preprocessing, auditory stimulus presentation, data normalization, brain activity source localization, and the quantification of auditory evoked fields (AEFs) across different sound intensity levels.

Prior to and following the ECT course, patients underwent MEG scans (see **Figure 1**). MEG recordings were captured using the Elektra Neuromag VectorView 306 system, which is equipped with 102 magnetometers and 204 planar gradiometers. To ensure accurate alignment, the MRIs were coregistered with scalp fiducial markers. While seated inside the MEG helmet, patients were exposed to a series of auditory tones at five different intensity levels: 55, 65, 75, 85, and 95dB. The tones were emitted through biauricular earbuds at a frequency of 2kHz, lasting 50ms each. The tones were presented in a random sequence, with interstimulus intervals of 1.2–2s; each intensity block comprising 22 tones, resulting in a total of 110 trials per intensity level.

Data analysis was performed with Brainstorm 3 (54), which is documented and freely available for download online under the GNU general public license (http://neuroimage.usc.edu/brainstorm). The MEG data was filtered using a 1–100Hz bandpass filter and a 60Hz notch filter to eliminate electrical line noise. Malfunctioning channels were identified and excluded. Artifacts arising from cardiac activity and eye blinks were removed via signal-space projection, and independent component analysis was used to eliminate other non-brain artifacts. Auditory events were defined for a time window from −100 to 500ms around the tone’s presentation. The data was normalized using the Z-transformation relative to the 100ms pre-stimulus baseline.

Cortical structures were derived from each subject’s MRI scans using FreeSurfer, and aligned with a standard brain atlas for cortical reconstruction. The head model for the forward model utilized the symmetric boundary element method (BEM) implemented in OpenMEEG, provided by the INRIA institute. This model established a computational link between the neuronal activity in the source space and the recorded MEG data in the sensor space, considering the conductive properties of head tissues. The inverse model, which infers neural activity from the MEG data, was computed using a data covariance matrix through the linearly constrained minimum variance (LCMV) beamforming technique, focusing on auditory evoked fields. Trial-averaged, source-level event-related fields (ERFs) were extracted from the bilateral primary auditory cortices. Finally, the LDAEP was calculated by evaluating the change in normalized ERF amplitude between the N100 and P200 components from the trial-averaged epochs. The LDAEP is represented by the slope of the linear regression line fitted to these data points.

### 2.3 Statistical analysis

We evaluated the distribution of our data for normality using the Shapiro–Wilk test. The tests indicated normality in the changes in the LDAEP slope (*W* = 0.86, *p* = .10), HDRS_24_ (*W* = 0.90, *p* = .26), and total RBANS scores (*W* = 0.84, *p* = .11). To investigate changes in LDAEP slopes, depression scores, and cognitive functioning scores before and after treatment, we employed paired *t*-tests. Additionally, we explored correlations among the pre-treatment and post-treatment LDAEP slopes, the degree of their changes, and the baseline, post-treatment, and changes in HDRS_24_ and RBANS scores. Initially, we attempted to include age and sex as covariates in a multiple linear regression model. However, given the small sample size of nine participants, which limited the statistical power and reliability of the estimates, and the lack of significant findings for age and sex, we decided to revert to simpler Pearson’s correlations.

## 3 Results

### 3.1 Demographics and clinical outcomes

The study involved nine participants, six of whom were female, with an age range from 50 to 78 years. The average age of the participants was 68.1 years with a standard deviation of 10.7 years, and six participants were 65 or older. Prior to receiving treatment, the average score on the 24-item Hamilton Depression Rating Scale (HDRS_24_) for these patients was 37.2 (standard deviation = 12.8). Additionally, the mean score on the total Repeatable Battery for the Assessment of Neuropsychological Status (RBANS) was 82.9 (19.4). Six of nine patients responded (> 50% reduction in HDRS_24_ from baseline) to ECT with an average post-ECT HDRS_24_ score of 9.1 ± 7.6 (*t*_8_ = 5.60, *p* < .001). We collected seven of the nine patients’ RBANS data, and found, on average, their cognitive functioning did not change with ECT (*t*_6_ = 0.36, *p* = .73). Demographics and clinical measures before and after ECT treatment are summarized in **Table 1**.

**Table 1:** Demographics and clinical measures before and after ECT treatment.

	Baseline	Post ECT	*t*	*p*
*N* (no. female)	9(6)	–	–	
Age, years, mean (SD)	68.1(10.7)	–	–	
LDAEP, mean (SD)	0.41(0.64)	0.75(0.74)	3.17	.013
HDRS_24_, mean (SD)	37.2(12.8)	9.1(7.6)	5.60	*< .*001
RBANS scores, mean (SD)				
Total	82.9(19.4)	84.4(18.0)	0.36	.73
Immediate memory	78.0(25.4)	83.1(24.2)	0.84	.44
Visuospatial/ Constructional	84.7(26.1)	90.7(17.7)	0.79	.46
Language	92.6(8.9)	89.7(8.0)	0.79	.46
Attention	92.4(14.7)	89.6(17.9)	1.05	.34
Delayed memory	85.3(22.1)	85.7(22.2)	0.08	.94

### 3.2 Change in LDAEP

**Figure 2:**
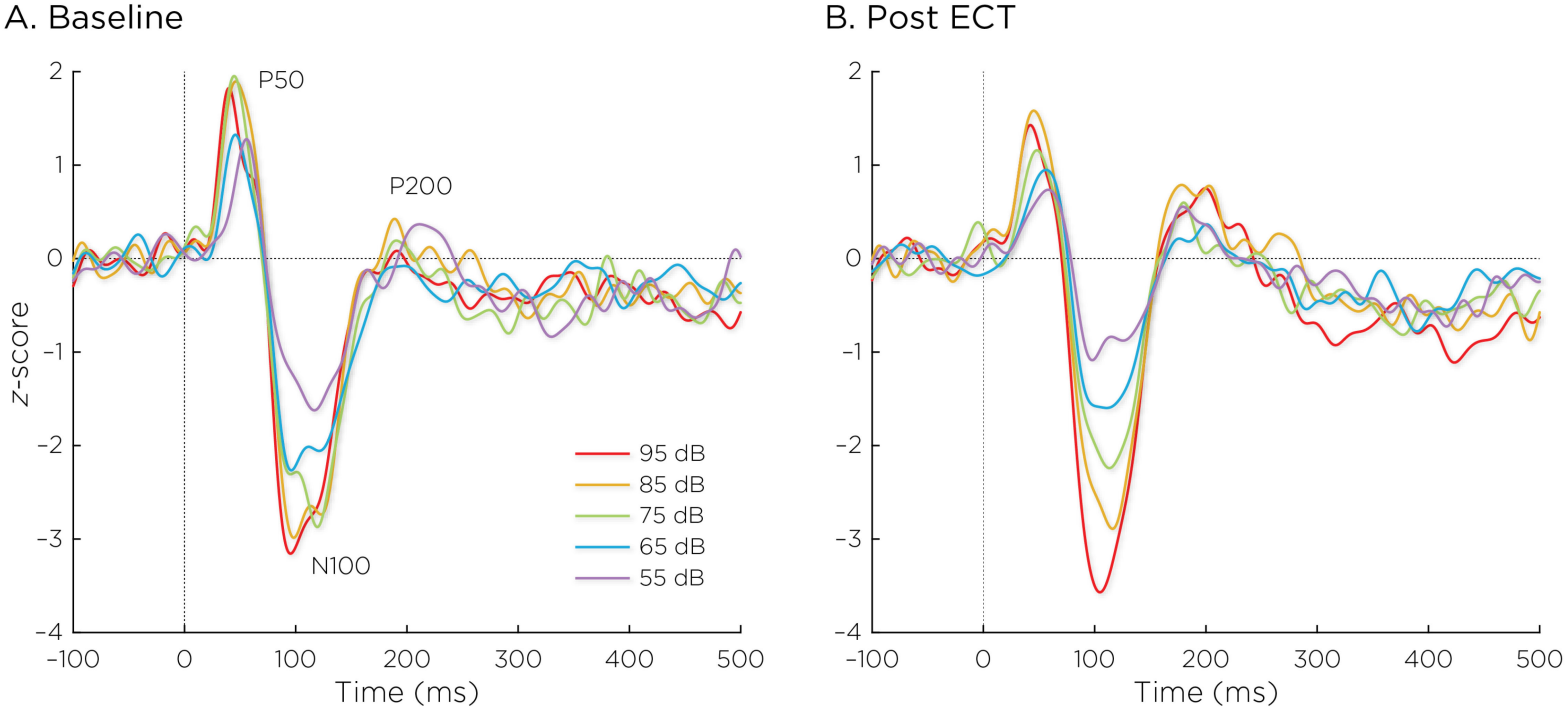
Auditory evoked fields **(A)** before and **(B)** after ECT treatment, with responses at sound pressure levels of 55–95dB. The change in normalized evoked field amplitude between the N100 and P200 (N1/P2) components of the trial-averaged epochs was calculated. The LDAEP is calculated as the slope of linear regression line that best fits the N1/P2 amplitudes at each sound pressure level.

**Figure 3:**
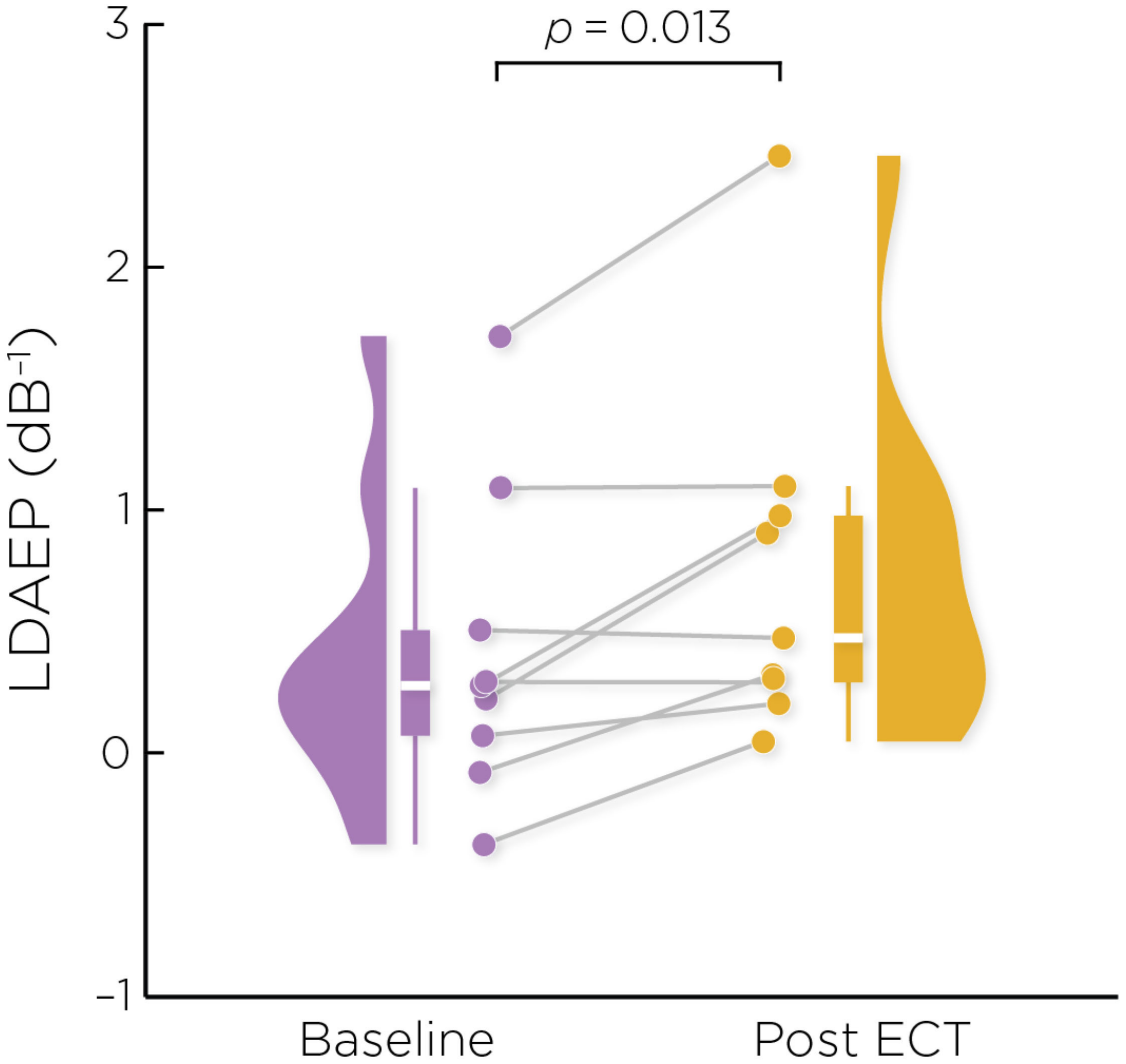
Individual changes in the pre- and post-ECT LDAEP slope measure. There was a significant increase post-ECT compared to baseline (Cohen’s *d* = 0.49, *t*_8_ = 3.17, *p* = .013).

**Figure 2** shows the auditory evoked fields before and after ECT treatment, with responses at varying stimulus loudness levels. The LDAEP slope significantly increased following ECT treatment from 0.41 ± 0.64 to 0.75 ± 0.74) (Cohen’s *d* = 0.49, *t*_8_ = 3.17, *p* = .013) (**Figure 3**).

### 3.3 Correlations between LDAEP and depression and cognition scores

The change in the LDAEP slope was not significantly correlated with baseline (*r* = −0.079, *p* = .84), post-treatment (*r* = 0.066, *p* = .87), or changes in HDRS_24_ (*r* = 0.101, *p* = .80). The pre-ECT LDAEP slope was not significantly correlated with baseline (*r* = −0.194, *p* = .62) nor changes in HDRS_24_ (*r* = 0.339, *p* = .37).

We focused on assessing correlations with RBANS total scores. The change in LDAEP slope was not significantly correlated with the baseline (*r* = 0.208, *p* = .66), post-treatment (*r* = 0.403, *p* = .37), or changes (*r* = 0.276, *p* = .55) in the RBANS total score. The pre-ECT LDAEP slope was significantly correlated with the baseline RBANS total score (*r* = 0.855, *p* = .014), but not correlated with changes in the RBANS total score (*r* = −0.352, *p* = .44).

## 4 Discussion

In this study, we used LDAEP as a cortical activity biomarker to monitor changes in neurotransmitter activity induced by ECT. Our initial hypothesis posited that ECT would mitigate symptoms of depression by boosting serotonergic neurotransmission, which would manifest as a reduced LDAEP, reflected by weakening of the response amplitude as a function of sound intensity levels. However, our findings revealed a significant increase in LDAEP post-ECT. Interestingly, the alterations in LDAEP did not correlate with changes in depression severity or cognitive performance.

The neurochemical underpinnings of LDAEP suggest that ECT should lead to a reduction in serotonergic tone within the primary auditory cortex, but this assumption is subject to debate. Studies on the relationship between LDAEP and serotonergic activity have harbored conflicting evidence and perspectives (21, 26, 55, 56). While numerous studies have found that the LDAEP is sensitive to acute changes in serotonergic activity, such as following administration of serotonergic-reuptake inhibitors, other studies have presented contrasting findings (57–63). A recent narrative review by Kangas et al. stated that LDAEP studies have generally yielded no consistent difference between depressed and non-depressed controls, though there appears to be a relationship with depression-subtypes (20). Given this context, a possible explanation for our results not supporting the original hypothesis could be that the LDAEP does not precisely mirror serotonergic tone in the primary auditory cortex.

Our findings did reveal a significant modulation of the LDAEP after ECT treatment. Intriguingly, the observed increase in LDAEP may indicate that ECT prompted a reduction in serotonergic receptor activity within the primary auditory cortex, particularly 5-HT1A and 5-HT2 receptors. This aligns with multiple studies demonstrating a reduction in 5-HT2 and 5-HT1A receptors in humans and non-human primates following ECT (41–43, 64, 65). The initial LDAEP studies in animal models bolster the plausibility of ECT-induced reduction in serotonergic receptors, in that a 5-HT1A agonist decreased the LDAEP and a 5-HT2A antagonist increased the LDAEP, suggesting that decreased serotonin receptor activity results in a strengthened LDAEP (13, 66). However, pre-clinical studies have largely found an upregulation of 5-HT1A and 5-HT2A receptors after electroconvulsive stimuli in animal models (45, 47, 67). This discrepancy underscores the need for further research to understand ECT’s impact on serotonergic receptor activity in humans more comprehensively.

Several alternative explanations as to why the LDAEP increased following ECT may be plausible. The LDAEP has been shown to co-vary with symptom severity in disorders such as ADHD, schizophrenia, and Parkinson’s disease, all of which are strongly linked to dopaminergic dysregulation (68–70). Given that ECT has been found to significantly alter dopaminergic neurotransmission (45, 71–74), one possible explanation for the heightened LDAEP could be ECT’s direct influence on dopamine receptor and transporter activities. Furthermore, considering that the LDAEP results from both excitatory and inhibitory post-synaptic potentials within the primary auditory cortex, the altered LDAEP could reflect changes in glutamatergic and GABAergic functions. Indeed, ECT has been shown to increase GABA concentration, normalize glutamate deficits, and alter excitation/inhibition ratios (28, 75–78). For instance, the administration of a glutamatergic NMDA antagonist has been reported to blunt LDAEP, suggesting that increased glutamatergic activity correlates with a heightened LDAEP (79). On the other hand, a study reported that the LDAEP is not associated with GABA levels (80). Further exploration of the relationships between ECT, auditory cortical activity, and excitatory and inhibitory neurotransmitters could yield valuable insights.

Beyond neurotransmitter effects, there is a consistent body of evidence indicating that ECT is associated with increased gray matter volume in the temporal lobes, including the superior temporal gyrus (5, 81–83), where the primary auditory cortex is situated. It is possible that neurotrophic effects are related to the increased LDAEP following ECT in this study. A recent meta-analysis concluded that ECT directly increases concentrations of brain-derived neurotrophic factor (BDNF) (84). Similarly, the LDAEP has been found to be significantly positively correlated with serum BDNF levels (85). One possibility is that ECT’s robust neuroplastic effects within the temporal lobe are related to the modulation of the LDAEP. Moreover, systematic reviews found that ECT has consistently been reported to decrease levels of inflammatory biomarkers, tumor necrosis factor alpha (TNF-*α*) and interleukin-6 (IL-6) (86, 87). Notably, one study demonstrated that the LDAEP was negatively correlated with TNF-α (88). This could suggest that a reduction in TNF-α might contribute to the LDAEP increase seen after ECT. Given these multifaceted biological interactions, further research is indeed warranted to unravel the complexities of ECT’s impact on the LDAEP and underlying neurobiological mechanisms.

Moreover, the primary auditory cortex is located within the superior temporal gyrus (STG). Significant changes in the LDAEP likely indicate changes in neural activity within parts of the STG anatomically and functionally connected to the PAC and potentially within the temporal lobe in general. For example, in a 2020 study, Pillai et al. found that the LDAEP was significantly correlated positively with the 5-HT1a receptor and negatively with 5-HTT throughout the temporal cortex (15). In the case of an altered LDAEP, far-reaching effects within the brain are likely given the temporal cortex’s pivotal role in the default mode network, social cognition network, and executive control network. Future work can employ techniques such as dynamic causal modeling to estimate how different brain regions interact during the processing of the auditory stimuli, thus allowing for more insightful delineation of the specific neural pathways involved in LDAEP.

In terms of cognitive performance, our study found that the pre-ECT LDAEP correlated with baseline RBANS total scores. However, change in LDAEP was not associated with changes in cognitive performance post-ECT. While the reasons for these findings warrant further investigation, our preliminary data does suggest a link between LDAEP and cognitive performance metrics.

Finally, it is crucial to acknowledge the limitations inherent in this pilot study. The small sample size of nine participants may not fully represent the broader patient population; these findings must be regarded as exploratory. It is important to recognize that the participants in this study are older adults, with six participants aged 65 or older. Age can influence clinical responses and ECT-induced biomarker changes. However, this study does not have the statistical power to assess the influence of age adequately. Future studies with larger and more diverse samples are needed to confirm our findings and better understand the impact of age and sex on ECT treatment efficacy and related biomarkers. Furthermore, the impact of the patients’ ongoing psychotropic medication on the LDAEP results also cannot be overlooked. The heterogeneous treatment responses and age can also influence the LDAEP outcome. Despite these limitations, this study provides meaningful insights into the changes in LDAEP following ECT and signals the importance of conducting larger-scale, more controlled research to elucidate these preliminary observations.

## 5 Conclusion

Contrary to our initial hypothesis, ECT paradoxically led to an increase in LDAEP, implying a reduction in serotonergic activity. Given the complex roles ECT plays in the brain’s neurochemistry and the multi-faceted nature of LDAEP as a biological marker, our results might not signal a straightforward suppression of serotonin. They could reflect compensatory adjustments in serotonergic receptor activity or broader changes encompassing other neurotransmitter systems, neuroplasticity, and neuro-immune interactions. This unexpected outcome opens avenues for multiple lines of inquiry: the intricate interplay between ECT and LDAEP, and how they might influence 1) the activity of serotonergic, dopaminergic, glutamatergic, and GABAergic receptors and transporters; 2) neuroplasticity and BDNF levels in the temporal cortex; and 3) levels of the pro-inflammatory cytokine TNF-α. Looking forward, further investigation is needed to validate the LDAEP as a biomarker of serotonergic neurotransmission and to elucidate ECT’s effect on serotonergic activity in the human brain.

## Data Availability

The raw data supporting the conclusions of this article will be made available by the authors, without undue reservation.

## Footnotes

1 Note that “LDAEP” traditionally refers to potentials recorded via EEG. For consistency with the literature, we retain this familiar nomenclature. However, it is important to note that in the context of this study, “LDAEP” denotes the magnetic fields measured by MEG rather than electrical potentials.

## References

[1] Bains N Abdijadid S. Major depressive disorder. Treasure Island, FL: StatPearls Publishing (2024). Available online at: http://www.ncbi.nlm.nih.gov/books/NBK559078/ (Accessed April 9, 2024). 32644504

[2] Hsieh MH. Electroconvulsive therapy for treatment-resistant depression. Prog Brain Res. (2023) 281:69–90. doi: 10.1016/bs.pbr.2023.01.004 37806717

[3] Subramanian S Lopez R Zorumski CF Cristancho P. Electroconvulsive therapy in treatment resistant depression. J Neurol Sci. (2022) 434:120095. doi: 10.1016/j.jns.2021.120095 34979372

[4] Deng ZD Robins PL Regenold W Rohde P Dannhauer M Lisanby SH. How electroconvulsive therapy works in the treatment of depression: is it the seizure, the electricity, or both? Neuropsychopharmacology. (2024) 49:150–62. doi: 10.1038/s41386-023-01677-2 PMC10700353 37488281

[5] Chen X Yang H Cui LB Li X. Neuroimaging study of electroconvulsive therapy for depression. Front Psychiatry. (2023) 14:1170625. doi: 10.3389/fpsyt.2023.1170625 37363178 PMC10289201

[6] Rojas M Ariza D Ortega A Riaño Garzón ME Chávez-Castillo M Pérez JL. Electroconvulsive therapy in psychiatric disorders: A narrative review exploring neuroendocrine-immune therapeutic mechanisms and clinical implications. Int J Mol Sci. (2022) 23:6918. doi: 10.3390/ijms23136918 35805923 PMC9266340

[7] Ousdal OT Brancati GE Kessler U Erchinger V Dale AM Abbott C. The neurobiological effects of electroconvulsive therapy studied through magnetic resonance: What have we learned, and where do we go? Biol Psychiatry. (2022) 91:540–9. doi: 10.1016/j.biopsych.2021.05.023 PMC8630079 34274106

[8] Maffioletti E Carvalho Silva R Bortolomasi M Baune BT Gennarelli M Minelli A. Molecular biomarkers of electroconvulsive therapy effects and clinical response: Understanding the present to shape the future. Brain Sci. (2021) 11:1120. doi: 10.3390/brainsci11091120 34573142 PMC8471796

[9] Belge JB Mulders P Van Diermen L Sienaert P Sabbe B Abbott CC. Reviewing the neurobiology of electroconvulsive therapy on a micro-, meso-, and macro-level. Prog Neuropsychopharmacol Biol Psychiatry. (2023) 127:110809. doi: 10.1016/j.pnpbp.2023.110809 37331685

[10] Cano M Camprodon JA. Understanding the mechanisms of action of electroconvulsive therapy: Revisiting neuroinflammatory and neuroplasticity hypotheses. JAMA Psychiatry. (2023) 80:643–4. doi: 10.1001/jamapsychiatry.2023.0728 37074712

[11] Delgado PL. Depression: The case for a monoamine deficiency. J Clin Psychiatry. (2000) 61 Suppl 6:7–11. 10775018

[12] Baldinger P Lotan A Frey R Kasper S Lerer B Lanzenberger R. Neurotransmitters and electroconvulsive therapy. J ECT. (2014) 30:116–21. doi: 10.1097/YCT.0000000000000138 24820941

[13] Juckel G Hegerl U Molnár M Csépe V Karmos G. Auditory-evoked potentials as indicator of brain serotonergic activity–first evidence in behaving cats. Biol Psychiatry. (1997) 41:1181–95. doi: 10.1016/s0006-3223(96)00240-5 9171909

[14] Lewis DA Campbell MJ Foote SL Morrison JH. The monoaminergic innervation of primate neocortex. Hum Neurobiol. (1986) 5:181–8. 3533864

[15] Pillai R Bartlett E Ananth M Zhu Z Yang J Hajcak G. Examining the underpinnings of loudness dependence of auditory evoked potentials with positron emission tomography. Neuroimage. (2020) 213:116733. doi: 10.1016/j.neuroimage.2020.116733 32169543 PMC7254571

[16] Juckel G Hegerl U Molnár M Csépe V Karmos G. Auditory evoked potentials reflect serotonergic neuronal activity–a study in behaving cats administered drugs acting on 5-HT1A autoreceptors in the dorsal raphe nucleus. Neuropsychopharmacology. (1999) 21:710–6. doi: 10.1016/S0893-133X(99)00074-3 10633476

[17] Buchsbaum M. Individual differences in stimulus intensity response. Psychophysiology. (1971) 8:600–11. doi: 10.1111/j.1469-8986.1971.tb00496.x 5116825

[18] Manjarrez G Hernandez E Robles A Hernandez J. N1/P2 component of auditory evoked potential reflect changes of the brain serotonin biosynthesis in rats. Nutr Neurosci. (2005) 8:213–8. doi: 10.1080/10284150500170971 16491646

[19] Hegerl U Gallinat J Juckel G. Event-related potentials. do they reflect central serotonergic neurotransmission and do they predict clinical response to serotonin agonists? J Affect Disord. (2001) 62:93–100. doi: 10.1016/s0165-0327(00)00353-0 11172876

[20] Kangas ES Vuoriainen E Lindeman S Astikainen P. Auditory event-related potentials in separating patients with depressive disorders and non-depressed controls: A narrative review. Int J Psychophysiol. (2022) 179:119–42. doi: 10.1016/j.ijpsycho.2022.07.003 35839902

[21] Roser P Kawohl W Juckel G. Chapter 20 - The loudness dependence of auditory evoked potentials as an electrophysiological marker of central serotonergic neurotransmission: Implications for clinical psychiatry and psychopharmacotherapy (Elsevier). In: Handbook of behavioral neuroscience, vol. 31 (Cambridge, MA: Academic Press), (2020). p. 361–74. doi: 10.1016/B978-0-444-64125-0.00020-7

[22] Pan W Pan J Zhao Y Zhang H Tang J. Serotonin transporter defect disturbs structure and function of the auditory cortex in mice. Front Neurosci. (2021) 15:749923. doi: 10.3389/fnins.2021.749923 34690685 PMC8527018

[23] Rao D Basura GJ Roche J Daniels S Mancilla JG Manis PB. Hearing loss alters serotonergic modulation of intrinsic excitability in auditory cortex. J Neurophysiol. (2010) 104:2693–703. doi: 10.1152/jn.01092.2009 PMC2997032 20884760

[24] Lee KKY Soutar CN Dringenberg HC. Gating of long-term potentiation (LTP) in the thalamocortical auditory system of rats by serotonergic (5-HT) receptors. Brain Res. (2018) 1683:1–11. doi: 10.1016/j.brainres.2018.01.004 29325855

[25] Steinberg LJ Underwood MD Bakalian MJ Kassir SA Mann JJ Arango V. 5-HT1A receptor, 5-HT2A receptor and serotonin transporter binding in the human auditory cortex in depression. J Psychiatry Neurosci. (2019) 44:294–302. doi: 10.1503/jpn.180190 31120232 PMC6710086

[26] O’Neill BV Croft RJ Nathan PJ. The loudness dependence of the auditory evoked potential (LDAEP) as an in *vivo* biomarker of central serotonergic function in humans: Rationale, evaluation and review of findings. Hum Psychopharmacol. (2008) 23:355–70. doi: 10.1002/hup.940 18421800

[27] Zhang M Siegle GJ. Linking affective and hearing sciences-affective audiology. Trends Hear. (2023) 27:23312165231208377. doi: 10.1177/23312165231208377 37904515 PMC10619363

[28] Keesom SM Hurley LM. Silence, solitude, and serotonin: Neural mechanisms linking hearing loss and social isolation. Brain Sci. (2020) 10:367. doi: 10.3390/brainsci10060367 32545607 PMC7349698

[29] Tang ZQ Trussell LO. Serotonergic modulation of sensory representation in a central multisensory circuit is pathway specific. Cell Rep. (2017) 20:1844–54. doi: 10.1016/j.celrep.2017.07.079 PMC5600294 28834748

[30] Papesh M Hurley L. Modulation of auditory brainstem responses by serotonin and specific serotonin receptors. Hear Res. (2016) 332:121–36. doi: 10.1016/j.heares.2015.11.014 26688176

[31] Christ M Michael N Hihn H Schüttke C Konrad C Baune BT. Auditory processing of sine tones before, during and after ECT in depressed patients by fMRI. J Neural Transm. (2008) 115:1199–211. doi: 10.1007/s00702-008-0036-5 18317681

[32] Zwanzger P Zavorotnyy M Diemer J Ruland T Domschke K Christ M. Auditory processing in remitted major depression: A long-term follow-up investigation using 3T-fMRI. J Neural Transm. (2012) 119:1565–73. doi: 10.1007/s00702-012-0871-2 22926663

[33] Zweerings J Zvyagintsev M Turetsky BI Klasen M König AA Roecher E. Fronto-parietal and temporal brain dysfunction in depression: A fMRI investigation of auditory mismatch processing. Hum Brain Mapp. (2019) 40:3657–68. doi: 10.1002/hbm.24623 PMC6865717 31081231

[34] Opitz B Schröger E von Cramon DY. Sensory and cognitive mechanisms for preattentive change detection in auditory cortex. Eur J Neurosci. (2005) 21:531–5. doi: 10.1111/j.1460-9568.2005.03839.x 15673452

[35] Cervantes-Ramírez V Canto-Bustos M Aguilar-Magaña D Pérez-Padilla EA Góngora-Alfaro JL Pineda JC. Citalopram reduces glutamatergic synaptic transmission in the auditory cortex *via* activation of 5-HT1A receptors. Neuroreport. (2019) 30:1316–22. doi: 10.1097/WNR.0000000000001366 31714483

[36] Timmermann C Spriggs MJ Kaelen M Leech R Nutt DJ Moran RJ. LSD modulates effective connectivity and neural adaptation mechanisms in an auditory oddball paradigm. Neuropharmacology. (2018) 142:251–62. doi: 10.1016/j.neuropharm.2017.10.039 29101022

[37] Ampuero E Cerda M Härtel S Rubio FJ Massa S Cubillos P. Chronic fluoxetine treatment induces maturation-compatible changes in the dendritic arbor and in synaptic responses in the auditory cortex. Front Pharmacol. (2019) 10:804. doi: 10.3389/fphar.2019.00804 31379577 PMC6650542

[38] Griskova I Dapsys K Andruskevicius S Ruksenas O. Does electroconvulsive therapy (ECT) affect cognitive components of auditory evoked P300? Acta Neurobiol Exp. (2005) 65:73–7. doi: 10.55782/ane-2005-1541 15794033

[39] Nurminen M Valkonen-Korhonen M Mervaala E Pääkkönen A Partanen J Viinamäki H. Enhanced attention-dependent auditory processing by electroconvulsive therapy in psychotic depression. J ECT. (2005) 21:19–24. doi: 10.1097/01.yct.0000158015.88677.bc 15791173

[40] Carhart-Harris RL Nutt DJ. Serotonin and brain function: A tale of two receptors. J Psychopharmacol. (2017) 31:1091–120. doi: 10.1177/0269881117725915 PMC5606297 28858536

[41] Yatham LN Liddle PF Lam RW Zis AP Stoessl AJ Sossi V. Effect of electroconvulsive therapy on brain 5-HT(2) receptors in major depression. Br J Psychiatry. (2010) 196:474–8. doi: 10.1192/bjp.bp.109.069567 20513859

[42] Lanzenberger R Baldinger P Hahn A Ungersboeck J Mitterhauser M Winkler D. Global decrease of serotonin-1A receptor binding after electroconvulsive therapy in major depression measured by PET. Mol Psychiatry. (2013) 18:93–100. doi: 10.1038/mp.2012.93 22751491 PMC3526726

[43] Strome E Clark C Zis A Doudet D. Electroconvulsive shock decreases binding to 5-HT2 receptors in nonhuman primates: an in *vivo* positron emission tomography study with [18F]setoperone. Biol Psychiatry. (2005) 57:1004–10. doi: 10.1016/j.biopsych.2005.01.025 15860341

[44] Saijo T Takano A Suhara T Arakawa R Okumura M Ichimiya T. Effect of electroconvulsive therapy on 5-HT1A receptor binding in patients with depression: a PET study with [11C]WAY 100635. Int J Neuropsychopharmacol. (2010) 13:785–91. doi: 10.1017/S1461145709991209 20067660

[45] Landau AM Alstrup AKO Noer O Winterdahl M Audrain H Møller A. Electroconvulsive stimulation differentially affects [11C]MDL100, 907 binding to cortical and subcortical 5HT2A receptors in porcine brain. J Psychopharmacol. (2019) 33:714–21. doi: 10.1177/0269881119836212 30887871

[46] Hvilsom AST Lillethorup TP Iversen P Doudet DJ Wegener G Landau AM. Cortical and striatal serotonin transporter binding in a genetic rat model of depression and in response to electroconvulsive stimuli. Eur Neuropsychopharmacol. (2019) 29:493–500. doi: 10.1016/j.euroneuro.2019.02.009 30826156

[47] Burnet PW Sharp T LeCorre SM Harrison PJ. Expression of 5-HT receptors and the 5-HT transporter in rat brain after electroconvulsive shock. Neurosci Lett. (1999) 277:79–82. doi: 10.1016/s0304-3940(99)00857-5 10624814

[48] Li XK Qiu HT. Current progress in neuroimaging research for the treatment of major depression with electroconvulsive therapy. World J Psychiatry. (2022) 12:128–39. doi: 10.5498/wjp.v12.i1.128 PMC8783162 35111584

[49] Jiang J Wang J Li C. Potential mechanisms underlying the therapeutic effects of electroconvulsive therapy. Neurosci Bull. (2016) 33:339–47. doi: 10.1007/s12264-016-0094-x PMC5567510 28032314

[50] Hari R Kaila K Katila T Tuomisto T Varpula T. Interstimulus interval dependence of the auditory vertex response and its magnetic counterpart: Implications for their neural generation. Electroencephalogr Clin Neurophysiol. (1982) 54:561–9. doi: 10.1016/0013-4694(82)90041-4 6181979

[51] Wyss C Foers F Kawohl W Arrubla J Vahedipour K Dammers J. Spatiotemporal properties of auditory intensity processing in multisensor MEG. Neuroimage. (2014) 102:465–73. doi: 10.1016/j.neuroimage.2014.08.012 25132019

[52] Abbott CC Lemke NT Gopal S Thomas RJ Bustillo J Calhoun VD. Electroconvulsive therapy response in major depressive disorder: a pilot functional network connectivity resting state fMRI investigation. Front Psychiatry. (2013) 4:10. doi: 10.3389/fpsyt.2013.00010 23459749 PMC3585433

[53] Fischl B Salat DH Busa E Albert M Dieterich M Haselgrove C. Whole brain segmentation: automated labeling of neuroanatomical structures in the human brain. Neuron. (2002) 33:341–55. doi: 10.1016/S0896-6273(02)00569-X 11832223

[54] Tadel F Baillet S Mosher JC Pantazis D Leahy RM. Brainstorm: A user-friendly application for MEG/EEG analysis. Comput Intell Neurosci. (2011) 2011:879716. doi: 10.1155/2011/879716 21584256 PMC3090754

[55] Ostermann J Uhl I Köhler E Juckel G Norra C. The loudness dependence of auditory evoked potentials and effects of psychopathology and psychopharmacotherapy in psychiatric inpatients. Hum Psychopharmacol. (2012) 27:595–604. doi: 10.1002/hup.2269 24446538

[56] Obermanns J Flasbeck V Steinmann S Juckel G Emons B. Investigation of the serotonergic activity and the serotonin content in serum and platelet, and the possible role of the serotonin transporter in patients with depression. Behav Sci. (2022) 12:178. doi: 10.3390/bs12060178 35735388 PMC9220674

[57] Uhl I Gorynia I Gallinat J Mulert C Wutzler A Heinz A. Is the loudness dependence of auditory evoked potentials modulated by the selective serotonin reuptake inhibitor citalopram in healthy subjects? Hum Psychopharmacol. (2006) 21:463–71. doi: 10.1002/hup.803 17029304

[58] Guille V Croft RJ O’Neill BV Illic S Phan KL Nathan PJ. An examination of acute changes in serotonergic neurotransmission using the loudness dependence measure of auditory cortex evoked activity: effects of citalopram, escitalopram and sertraline. Hum Psychopharmacol. (2008) 23:231–41. doi: 10.1002/hup.922 18196604

[59] Oliva J Leung S Croft RJ O’Neill BV O’Kane J Stout J. The loudness dependence auditory evoked potential is insensitive to acute changes in serotonergic and noradrenergic neurotransmission. Hum Psychopharmacol. (2010) 25:423–7. doi: 10.1002/hup.1133 20589921

[60] O’Neill BV Guille V Croft RJ Leung S Scholes KE Phan KL. Effects of selective and combined serotonin and dopamine depletion on the loudness dependence of the auditory evoked potential (LDAEP) in humans. Hum Psychopharmacol. (2008) 23:301–12. doi: 10.1002/hup.926 18213738

[61] Nathan PJ Segrave R Phan KL O’Neill B Croft RJ. Direct evidence that acutely enhancing serotonin with the selective serotonin reuptake inhibitor citalopram modulates the loudness dependence of the auditory evoked potential (LDAEP) marker of central serotonin function. Hum Psychopharmacol. (2006) 21:47–52. doi: 10.1002/hup.740 16317803

[62] Wutzler A Winter C Kitzrow W Uhl I Wolf RJ Heinz A. Loudness dependence of auditory evoked potentials as indicator of central serotonergic neurotransmission: simultaneous electrophysiological recordings and in *vivo* microdialysis in the rat primary auditory cortex. Neuropsychopharmacology. (2008) 33:3176–81. doi: 10.1038/npp.2008.42 18463629

[63] Simmons JG Nathan PJ Berger G Allen NB. Chronic modulation of serotonergic neurotransmission with sertraline attenuates the loudness dependence of the auditory evoked potential in healthy participants. Psychopharmacology. (2011) 217:101–10. doi: 10.1007/s00213-011-2265-9 21465243

[64] Plein H Berk M. Changes in the platelet intracellular calcium response to serotonin in patients with major depression treated with electroconvulsive therapy: State or trait marker status. Int Clin Psychopharmacol. (2000) 15:93–8. doi: 10.1097/00004850-200015020-00005 10759340

[65] Ishihara K Sasa M. Mechanism underlying the therapeutic effects of electroconvulsive therapy (ECT) on depression. Jpn J Pharmacol. (1999) 80:185–9. doi: 10.1254/jjp.80.185 10461762

[66] Juckel G Gallinat J Riedel M Sokullu S Schulz C Möller HJ. Serotonergic dysfunction in schizophrenia assessed by the loudness dependence measure of primary auditory cortex evoked activity. Schizophr Res. (2003) 64:115–24. doi: 10.1016/s0920-9964(03)00016-1 14613676

[67] Vetulani J Lebrecht U Pilc A. Enhancement of responsiveness of the central serotonergic system and serotonin-2 receptor density in rat frontal cortex by electroconvulsive treatment. Eur J Pharmacol. (1981) 76:81–5. doi: 10.1016/0014-2999(81)90012-1 7318923

[68] Wyss C Hitz K Hengartner MP Theodoridou A Obermann C Uhl I. The loudness dependence of auditory evoked potentials (LDAEP) as an indicator of serotonergic dysfunction in patients with predominant schizophrenic negative symptoms. PloS One. (2013) 8:e68650. doi: 10.1371/journal.pone.0068650 23874705 PMC3709903

[69] Pauletti C Mannarelli D Locuratolo N Maffucci A Currà A Marinelli L. Serotonergic central tone in parkinson’s disease with fatigue: Evidence from the loudness dependence of auditory evoked potentials (ldaep). Neurosci Lett. (2021) 764:136242. doi: 10.1016/j.neulet.2021.136242 34509567

[70] Park E Park YM Lee SH Kim B. The loudness dependence of auditory evoked potentials is associated with the symptom severity and treatment in boys with attention deficit hyperactivity disorder. Clin Psychopharmacol Neurosci. (2022) 20:514–25. doi: 10.9758/cpn.2022.20.3.514 PMC9329111 35879036

[71] Saijo T Takano A Suhara T Arakawa R Okumura M Ichimiya T. Electroconvulsive therapy decreases dopamine D2 receptor binding in the anterior cingulate in patients with depression: A controlled study using positron emission tomography with radioligand [11C]FLB 457. J Clin Psychiatry. (2010) 71:793–8. doi: 10.4088/JCP.08m04746blu 20021995

[72] Strome EM Zis AP Doudet DJ. Electroconvulsive shock enhances striatal dopamine D1 and D3 receptor binding and improves motor performance in 6-OHDA-lesioned rats. J Psychiatry Neurosci. (2007) 32:193–202. 17476366 PMC1863551

[73] Masuoka T Tateno A Sakayori T Tiger M Kim W Moriya H. Electroconvulsive therapy decreases striatal dopamine transporter binding in patients with depression: A positron emission tomography study with [18F]FE-PE2I. Psychiatry Res Neuroimaging. (2020) 301:111086. doi: 10.1016/j.pscychresns.2020.111086 32464340

[74] Okamoto T Yoshimura R Ikenouchi-Sugita A Hori H Umene-Nakano W Inoue Y. Efficacy of electroconvulsive therapy is associated with changing blood levels of homovanillic acid and brain-derived neurotrophic factor (BDNF) in refractory depressed patients: a pilot study. Prog Neuropsychopharmacol Biol Psychiatry. (2008) 32:1185–90. doi: 10.1016/j.pnpbp.2008.02.009 18403081

[75] Ciranna L. Serotonin as a modulator of glutamate- and GABA-mediated neurotransmission: implications in physiological functions and in pathology. Curr Neuropharmacol. (2006) 4:101–14. doi: 10.2174/157015906776359540 PMC2430669 18615128

[76] Zhang J Narr KL Woods RP Phillips OR Alger JR Espinoza RT. Glutamate normalization with ECT treatment response in major depression. Mol Psychiatry. (2013) 18:268–70. doi: 10.1038/mp.2012.46 PMC3896297 22565784

[77] Sanacora G Mason GF Rothman DL Hyder F Ciarcia JJ Ostroff RB. Increased cortical GABA concentrations in depressed patients receiving ECT. Am J Psychiatry. (2003) 160:577–9. doi: 10.1176/appi.ajp.160.3.577 12611844

[78] Esel E Kose K Hacimusalar Y Ozsoy S Kula M Candan Z. The effects of electroconvulsive therapy on GABAergic function in major depressive patients. J ECT. (2008) 24:224–8. doi: 10.1097/YCT.0b013e31815cbaa1 18562944

[79] Teichert T. Loudness- and time-dependence of auditory evoked potentials is blunted by the NMDA channel blocker MK-801. Psychiatry Res. (2017) 256:202–6. doi: 10.1016/j.psychres.2017.06.047 PMC5723527 28645081

[80] Wyss C Tse DHY Boers F Shah NJ Neuner I Kawohl W. Association between cortical GABA and loudness dependence of auditory evoked potentials (LDAEP) in humans. Int J Neuropsychopharmacol. (2018) 21:809–13. doi: 10.1093/ijnp/pyy056 PMC6119294 29917080

[81] Deng ZD Argyelan M Miller J Quinn DK Lloyd M Jones TR. Electroconvulsive therapy, electric field, neuroplasticity, and clinical outcomes. Mol Psychiatry. (2022) 27:1676–82. doi: 10.1038/s41380-021-01380-y PMC9095458 34853404

[82] Argyelan M Oltedal L Deng ZD Wade B Joanlanne BAM Sanghani S. Electric field causes volumetric changes in the human brain. Elife. (2019) 8:e49115. doi: 10.7554/eLife.49115 31644424 PMC6874416

[83] Pirnia T Joshi SH Leaver AM Vasavada M Njau S Woods RP. Electroconvulsive therapy and structural neuroplasticity in neocortical, limbic and paralimbic cortex. Transl Psychiatry. (2016) 6:e832. doi: 10.1038/tp.2016.102 27271858 PMC4931600

[84] Pelosof R dos Santos LA Farhat LC Gattaz WF Talib L Brunoni AR. BDNF blood levels after electroconvulsive therapy in patients with mood disorders: An updated systematic review and meta-analysis. World J Biol Psychiatry. (2023) 24:24–33. doi: 10.1080/15622975.2022.2058083 35332840

[85] Park YM Lee BH Um TH Kim S. Serum BDNF levels in relation to illness severity, suicide attempts, and central serotonin activity in patients with major depressive disorder: A pilot study. PloS One. (2014) 9:e91061. doi: 10.1371/journal.pone.0091061 24663244 PMC3963843

[86] Gay F Romeo B Martelli C Benyamina A Hamdani N. Cytokines changes associated with electroconvulsive therapy in patients with treatment-resistant depression: A meta-analysis. Psychiatry Res. (2021) 297:113735. doi: 10.1016/j.psychres.2021.113735 33497973

[87] Desfossés CY Peredo R Chabot A Carmel JP Tremblay PM Mérette C. The pattern of change in depressive symptoms and inflammatory markers after electroconvulsive therapy: A systematic review. J ECT. (2021) 37:291–7. doi: 10.1097/YCT.0000000000000782 34294652

[88] Lee BH Park YM Lee SH Shim M. Serum levels of tumor necrosis factor-α and loudness dependence of auditory evoked potentials at pretreatment and posttreatment in patients with major depressive disorder. Brain Sci. (2019) 9:253. doi: 10.3390/brainsci9100253 31561419 PMC6826742

